# Cost-Effectiveness Analysis of Screening for and Managing Identified Hypertension for Cardiovascular Disease Prevention in Vietnam

**DOI:** 10.1371/journal.pone.0155699

**Published:** 2016-05-18

**Authors:** Thi-Phuong-Lan Nguyen, E. Pamela Wright, Thanh-Trung Nguyen, C. C. M. Schuiling-Veninga, M. J. Bijlsma, Thi-Bach-Yen Nguyen, M. J. Postma

**Affiliations:** 1 University of Groningen, Department of Pharmacy, Unit of PharmacoEpidemiology & PharmacoEconomics, Groningen, the Netherlands; 2 Medical Committee Netherlands-Vietnam, Amsterdam, The Netherlands; 3 Thai Nguyen University of Medicine and Pharmacy, Thai Nguyen, Vietnam; 4 Department of Health economic, Ha Noi University of Medicine, Ha Noi, Vietnam; 5 Institute for Science in Healthy Aging & healthcaRE (SHARE), University Medical Center Groningen (UMCG), Groningen, The Netherland; 6 Department of Epidemiology, UMCG, Groningen, The Netherlands; University of Miami School of Medicine, UNITED STATES

## Abstract

**Objective:**

To inform development of guidelines for hypertension management in Vietnam, we evaluated the cost-effectiveness of different strategies on screening for hypertension in preventing cardiovascular disease (CVD).

**Methods:**

A decision tree was combined with a Markov model to measure incremental cost-effectiveness of different approaches to hypertension screening. Values used as input parameters for the model were taken from different sources. Various screening intervals (one-off, annually, biannually) and starting ages to screen (35, 45 or 55 years) and coverage of treatment were analysed. We ran both a ten-year and a lifetime horizon. Input parameters for the models were extracted from local and regional data. Probabilistic sensitivity analysis was used to evaluate parameter uncertainty. A threshold of three times GDP per capita was applied.

**Results:**

Cost per quality adjusted life year (QALY) gained varied in different screening scenarios. In a ten-year horizon, the cost-effectiveness of screening for hypertension ranged from cost saving to Int$ 758,695 per QALY gained. For screening of men starting at 55 years, all screening scenarios gave a high probability of being cost-effective. For screening of females starting at 55 years, the probability of favourable cost-effectiveness was 90% with one-off screening. In a lifetime horizon, cost per QALY gained was lower than the threshold of Int$ 15,883 in all screening scenarios among males. Similar results were found in females when starting screening at 55 years. Starting screening in females at 45 years had a high probability of being cost-effective if screening biannually was combined with increasing coverage of treatment by 20% or even if sole biannual screening was considered.

**Conclusion:**

From a health economic perspective, integrating screening for hypertension into routine medical examination and related coverage by health insurance could be recommended. Screening for hypertension has a high probability of being cost-effective in preventing CVD. An adequate screening strategy can best be selected based on age, sex and screening interval.

## Introduction

Similar to the trend in global burden of disease, hypertension is a leading cause of cardiovascular disease (CVD) in Vietnam [1, 2]. According to Vietnam data in the Global Burden of Disease (GBD) report in 2013, cerebrovascular disease accounted for 9.7% of total disability adjusted life years (DALYs), of which hypertension contributed 57%. Ischemic heart disease (IHD) accounted for 2.4% of total DALYs, 51% of which is attributed to hypertension [2]. Treatment of hypertension is known to be effective in reducing the burden of CVD [3, 4]. A high prevalence of undiagnosed hypertension is an obstacle to treatment and prevention of complications. Globally, the prevalence of undiagnosed hypertension is 53% [5]; Vietnam is similar at 52% [6].

To increase knowledge and awareness of hypertension, early detection by measuring blood pressure (BP) is recommended [1]. Several studies on screening for hypertension and treatment in populations at risk, because of history of CVD, high cholesterol, diabetes or age, have demonstrated the cost-effectiveness of this approach in preventing cardiovascular or kidney disease [7–11]. Although there are other risk factors for CVD such as diabetes and high cholesterol, high blood pressure (HBP) may be considered the logical first focus, with age and sex as obvious potentially key guiding factors to the hypertension risk. Notably, HBP as an essential major CVD risk factor is incorporated into all risk predictors, potentially making BP screening the most essential factor for risk assessment in general.

WHO recommends that all adults check their BP regularly [1], either annually or every two years, depending on exact previous levels of BP [12]. However, in a limited-resource setting like Vietnam, it is not possible to screen everyone and it would be potentially too expensive to start screening for hypertension from 18 years of age. After many years of a focus on infectious diseases and safe motherhood, the Ministry of Health is currently developing plans to deal with non-communicable diseases, which have been increasing in the past decades. The Ministry of Health in Vietnam would like to integrate screening for hypertension into routine medical examination at all levels, especially at community health stations (CHS), and has proposed that these services will be covered by health insurance [13]. Therefore, policymakers and planners now urgently need evidence to guide the choices in their guidelines and recommendations. For example, policy makers may plan to postpone screening for hypertension to an older age when risk is higher, for example from the age of 40 [13]. Up to now, however, there is little evidence on the cost-effectiveness of screening for hypertension in developing countries. Previous studies have not provided sufficient evidence on the best screening strategies regarding screening intervals, or specific ages and sexes to be targeted. Information on the most effective strategies for low resource settings is scarce.

To meet that demand, we conducted a study to quantify quality-adjusted life years (QALYs) and incremental cost-effectiveness of the following approaches: (1) no screening; (2) one-off screening; (3) screening every two years;(4) annual screening; and (5) screening in combination with increased coverage of treatment in both sexes and different ages.

## Methods

### Model

**Fig 1:**
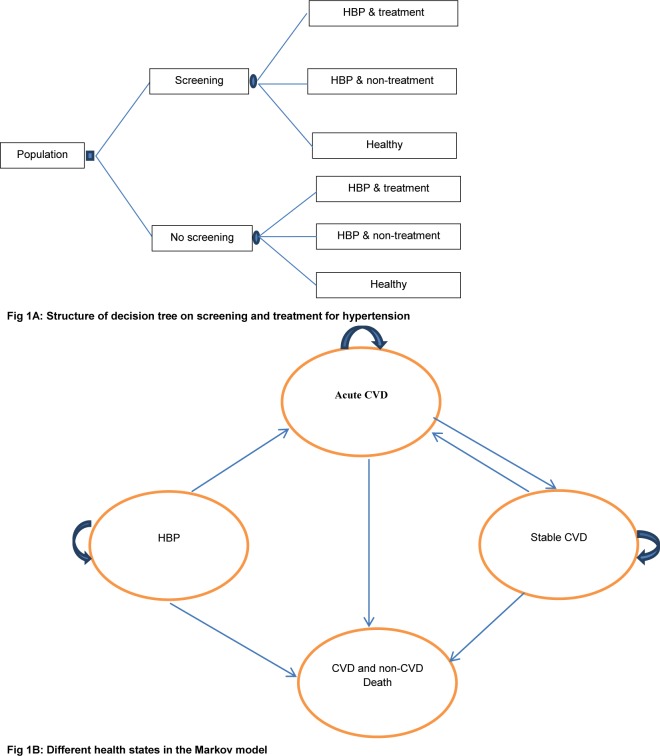
Decision tree and Markov model for estimating cost-effectiveness of screening for hypertension. Notes: HBP: high blood pressure; CVD: cardiovascular disease. Patients start in the initial hypertension state. Patients can remain in this state or move to either acute CVD or CVD/non-CVD death. From the acute CVD state, patients can move to stable CVD or CVD/non-CVD death state, or may experience recurrent CVD events. From the stable CVD state, patients may stay in the same health state, or they may have a recurrence of CVD or can move to CVD/non-CVD death.

The model combines a decision tree with a Markov model to estimate the cost-effectiveness of screening compared with non-screening for hypertension. Various intervals for screening (one-off, annually, biannually, biannually until 55 or 60 years old and then annually until death) and varying ages to start screening (35 or 45 or 55 years old), during a one-year cycle were applied and are presented in Table 1. The description of screening strategies is presented in the supporting material (S1 Table). In the model, for either screening or non-screening scenarios, people are divided into three groups: treated hypertension, untreated hypertension and healthy (non-hypertensive). In the Markov model, patients start in the initial hypertension state. Patients can remain in this state or move to either acute CVD or CVD/non-CVD death. At the end of each cycle in the acute CVD state, patients can move to stable CVD or CVD/non-CVD death state, or may experience recurrent CVD events and then stay in the same state. From the stable CVD state, patients can experience CVD/non-CVD death or stay in the same health state, or they may have a recurrence of CVD and move to acute CVD. Fig 1 illustrates the complete model.

**Table 1:** Intervention scenarios and time horizon.

Scenario	Description
	***1. 1*. *Ten-years horizon***
No	No screening, treatment
One-off	One-off screening in the first year, treatment
E1	Annual screening and treatment
E2	Biannual screening, treatment
E1&T.20%	Annual screening and increase coverage of treatment by 20%
E2&T.20%	Biannual screening and increase coverage of treatment by 20%
	***1. 2*. *Lifetime horizon***
No	No screening, treatment
E1	Annual screening, treatment
E2	Biannual screening, treatment
E2 until 55+ E1	Biannual screening until 55 years, then annual screening until death and treatment
E2 until 60+ E1	Biannual screening until 60 years, then annual screening until death and treatment
E1&T.20%	Annual screening and increase coverage of treatment by 20%
E2&T.20%	Biannual screening and increase coverage of treatment by 20%

**Notes**: 48%, 62% treatment among hypertension were applied in this study in male and female, respectively

We defined a composite CVD-outcome that included myocardial infarction (ICD-10 code I21) and cerebrovascular diseases (ICD-10 codes I60 to I66). Data on the incidence of myocardial infarction (MI) and of cerebrovascular disease in Vietnam were not available to weight the overall CVD. To overcome this limitation, data from a meta-analysis among hypertensive patients in an Asian population were used to design a composite CVD-outcome comprising 78% stroke (cerebrovascular diseases) and 22% MI [3]. Notably, this distribution was used to weight relative risk, costs and utilities in the overall composite CVD. The calculation of the composite CVD-outcome is presented in the S1 Dataset.

### Screening and hypertension management

We previously conducted community screening for hypertension among currently untreated, undiagnosed adults aged 35–64 years, measuring BP during two visits [14]. Individual cases with BP in the hypertensive range were confirmed by a doctor at the CHS. The details of this field work were described previously [14]. For the non-screening scenario/current practice, patients are assumed to visit the CHS for diagnosis and management when they have symptoms of hypertension.

Diagnosed patients in both the screening and non-screening scenarios were assumed to be receiving treatment for hypertension at the CHS. Antihypertensive drugs would be prescribed according to the Ministry of Health guidelines [15]. We assumed that patients with CVD were referred to a hospital for treatment.

### Prevalence and incidence of hypertension

In the screening scenario, total prevalence, stratified according to different age groups and sex, was quantified by prevalence of hypertension detected by screening plus prevalence diagnosed by symptoms. Prevalence of current treatment, after diagnosis through symptoms, was recalculated from a national survey on a sex-specific basis [6]. Among hypertensive patients, we estimated the prevalence of patients going or not going into treatment in accordance with a previous study [6]. The number of patients in the group going into treatment was aggregated with those currently under treatment, and the number of hypertensive patients detected by screening multiplied by the percentage on treatment among diagnosed patients. Coverage of treatment reflects the percentage of diagnosed patients who received treatment, additionally assuming that patients classified as on treatment strictly comply with therapy. Although in reality not all patients may be strictly adherent, we did not have reliable data to use as inputs in our analysis. Also, we could not find appropriate evidence on, for example, the association of adherence level and cardiovascular events, particularly in stroke and myocardial infarction events, in developing or Asian countries.

We also updated the data on annual incidence of hypertension in the model, extracting it from a previous study in a Vietnamese population [16]. The annual incidence was converted into an annual transition probability [17]. Although we expect that the probability is age and sex dependent, due to limited data, we had to assume that the probability remains constant with age and sex.

### Transition probabilities and relative risks

Probabilities for transitions between health states were extracted from previous studies. We started with a known population, for which data on age, sex, BP, cholesterol level and smoking status were available. The Asia Cardiovascular Risk Prediction model was applied to estimate the cumulative eight-year CVD risk (fatal CVD, MI, cerebrovascular diseases) for hypertensive patients [14, 18], subsequently converted into annual probabilities [17]. Notably, as mentioned above, age, sex, BP, smoking, and cholesterol level were used to predict CVD risk [18]. These data were extracted from our fieldwork because we could not access data from the national survey [14]. Probabilities were classified into two categories: fatal CVD and non-fatal CVD. The weighting scale for this measurement was re-calculated from individual studies of a meta-analysis in an Asian population for non-treatment groups (reference group) [3]. In the end, only age and sex were applied in the transition probabilities. To get equivalence of the coefficients for age and sex as in a model including cholesterol, BP level and smoking status, we fitted the transition probability CVD model with the grand-mean-centred predictors for BP, cholesterol level and smoking status [19, 20]. In addition, when there was no evidence for history of CVD among hypertensive patients, we assumed that they had no history of CVD at the time of data entry. If they did have CVD history, they would still have the same probability of transition from hypertension to acute CVD or CVD death as patients with no history of CVD [21].

The transition probability of acute to fatal CVD was calculated from individual studies of a meta-analysis among an Asian population for the non-treatment group [3]. We assumed this transition probability was stable for different ages and sexes.

The probability of recurrent CVD events of patients at the end of the acute or stable state may be expected to be higher than for subjects without a history of CVD events. However, there was no appropriate equation nor data to estimate the probability for those patients, so we assumed that the annual transition probability of patients with a history of CVD was the same as for those without CVD, when they were of the same age and sex.

Data from the Vietnam Life Table 2013 was used to quantify the transition probability from any health state to crude death (death due to all causes) [22] and to separate transition probability of non-(composite) CVD from crude death. The weighting scale for this estimate was re-calculated from a study on CVD mortality in Vietnam, assuming that the rate of death in CVD and non-CVD was stable by age [23] (possibly overestimated because CVD death may be from other heart disease).

Relative risks (RRs) of acute CVD and CVD death during treatment were also estimated based on individual studies of a meta-analysis among an Asian population [3, 24]. In the base case, we did not consider age-and sex-dependence for these RRs. Furthermore, results of the meta-analysis revealed no significantly different reductions in acute MI and stroke events, comparing different classes of antihypertensive drugs under similar BP control.

### Costs

Direct costs were quantified from the health service perspective, integrating four components: screening for hypertension, annual hypertension treatment, acute CVD treatment, and stable CVD treatment. All costs were calculated in international dollars (Int $) for the year 2013. To convert VND to Int $, we divided the amount in VND by the Purchasing Power Parity (PPP) exchange rate (in this case: 7,546.6) [25]. The discount rate for costs was 3% in the base case.

Screening costs were estimated from various sources. During fieldwork, village health workers and medical students measured people’s BP in communities, making it difficult to quantify the costs of these measurements in real-life practice. We made an assumption that each patient visited the CHS three times to be screened for hypertension, at 10 minutes per visit. The total cost of screening per person equalled the number of visits multiplied by the cost per primary care visit, taken from a previous study [26, 27].

Total cost of annual hypertension treatment per person was the sum of drug costs and CHS visits per month multiplied by twelve. Drug cost per month was obtained by multiplying number of pills on prescriptions for 338 patients treated at CHS, by prices extracted from an international drug price indicator guide 2013 [28], adding 30% for cost of transportation and distribution [26, 29]. Monthly visits to CHS were allocated 20 minutes on average; cost for visits were taken from a citation in a previous study [26, 27].

Costs of acute and stable CVD treatment were extracted from the database of Thai Nguyen Hospital, as described previously [30]. All patients with the relevant ICD codes, either code I21 or codes from I60 to I66, were included in the study. Treatment cost of the first and following years after the first acute MI event was calculated based on expert opinion. We applied a proportion of 68.5% patients with a first acute MI who had percutaneous coronary intervention or coronary artery bypass surgery [31] in the first-year treatment. We also assumed that there was no specific treatment or rehabilitation for stroke patients after acute events, which is common in Vietnam, where rehabilitation and long-term care is done by family members.

### Health utilities

Quality of life weights (utilities) for healthy, hypertensive, acute CVD and stable CVD cases were applied in the model. From published studies, we weighted utilities of all health states to the same scale of SF-6D. For example, utility of the general population in Vietnam was 0.88 by EQ-5D measurement [32]; subsequently applying the weighting scale between EQ-5D and SF-6D [33] identified utilities in SF-6D at 0.93. Health utility of hypertension was measured by SF-6D in Vietnam, assuming that utility is equal between treated and non-treated patients [34]. Heath utility of hypertension was also used to weight for MI which was not yet available in Vietnam. The health utility of stable MI was extracted from a Korean study and weighted for Vietnam, using a ratio of utility for hypertension patients between Korean and Vietnamese [35]. Health utility of stable stroke was extracted from a previous study in Vietnam [34]. Utility for the acute state was weighted from the stable state according to the ratio between acute and stable MI and stroke cited previously [34, 36, 37]. We did not discount utility in the base case.

### Base case and Sensitivity analysis

Cost-effectiveness was calculated in the base case and sensitivity analysis. Screening scenarios were combined with assumed increased levels of treatment among diagnosed hypertension (at 20% increase compared to baseline). Probabilistic sensitivity analysis in 5,000 repetitions of a Monte Carlo simulation examined the uncertainty of input parameters; Table 2 shows input values and distributions. Gamma distributions were applied for costs, beta distributions for health utilities, lognormal distributions for RRs and beta distributions for transition probabilities. We employed Cholesky decomposition to provide correlated draws, generated from a parameter’s multivariate normal distribution for transition probability of hypertension to death and acute CVD [17].

**Table 2:** Base-case model inputs and distribution.

Variables	Data	Distribution	Sources
Prevalence of HBP	5% to 41% (age and sex dependent)	Fixed	Re-calculation [6, 14]
Prevalence of HBP detected by screening	2.8% to 29.7% (age and sex dependent)	Fixed	Re-calculation [6, 14]
Prevalence of HBP not detected by screening	2.2% to 15.3% (age and sex dependent)	Fixed	Re-calculation [6]
Rate of going to treat among aware hypertensives	62%, 48% in female and male, respectively	Fixed	Re-calculation [6]
One-year transition probability from healthy to hypertension	0.0065 or 0.0164 in female and male, respectively	Beta	Re-calculation [16, 17]
One-year transition probability from HBP to non-fatal CVD	Age and sex dependent; Constant: (-9.54); Coefficients for age: 0.07; Coefficients for sex: 0.55	Cholesky	Re-calculation from ASIA CVD prediction model [3, 14, 17]
One-year transition probability from HBP to fatal CVD	Age and sex dependent; Constant: (-10.67); Coefficients for age: 0.07; Coefficients for sex: 0.55	Cholesky	Re-calculation [3, 14, 17]
One-year transition probability from acute CVD to death in the non-treatment group	0.008	Beta	[3, 17]
Cost of screening for hypertension	6.05	Gamma	Calculation based on cost of primary care reported in previous study [26]
Cost of HBP treatment in the community	70.82	Gamma	Calculation based on prescriptions at CHSs and [27]
Cost of acute CVD and treatment first year	3,723.24	Gamma	Calculation based on database of Thai Nguyen general hospital, Vietnam and expert’s opinion
Cost of stable CVD treatment in followed year	79.39	Gamma	Calculation based in an expert’s opinions
Utility in healthy state	0.93	Beta	Re-calculation [32, 33]
Utility in HBP-state	0.74 or 0.71 in male and female, respectively	Beta	[34]
Utility in acute CVD-state	0.67	Beta	Re-calculation [3, 34–37]
Utility in stable CVD-state	0.72 or 0.71 in male and female, respectively	Beta	Re-calculation [3, 34, 35]
Relative risk HBP to acute CVD	0.72	Lognormal	Re-calculation [3, 24]
Relative risk CVD-death	0.82	Lognormal	Re-calculation [3, 24]

Univariate sensitivity analysis was undertaken to examine the robustness of our model assumptions and data sources. This analysis included ± 25% of the transition probability from BP to fatal and non-fatal CVD and costs of screening, BP treatment and CVD treatment. Also, reducing health utilities of CVD by 10% and 20% and 1% or 3% discounting of QALYs was investigated.

For the purpose of international comparison, health utilities of MI and stroke were also based on disabilities extracted from the GBD 2010 study [38] and then weighted for health utilities of acute and stable CVD to quantify the effectiveness of the intervention. As disability weights of hypertension and healthy were not available at international level, they are weighted based on the values of the base case and these respective values from the GBD study. Notably, utility in acute CVD-state, stable CVD-state, healthy state and HBP-state were 0.685, 0.717, 0.957 and 0.835, respectively [38, 39].

The HBP prevalence of the national survey was higher than in our survey, therefore HBP prevalence according to the national survey was analyzed in a separate scenario. A key point for applying HBP prevalence from the national survey in a scenario analysis and not in the base case was that BP in the national survey was measured during one visit while in our study it was done on two different occasions, with a decrease in BP prevalence from 20.5% at the first visit to 12.3% in the second visit. We feel our survey is more likely to be accurate than the national survey. Furthermore, we suppose that the prevalence of hypertension in urban settings may be higher than in rural areas, as found in the national survey conducted in both rural and urban areas.

A meta-analysis report showed that the RR reduction of CVD is associated with age [4]. Lacking data in our setting, we conducted a scenario analysis which took the RR reduction of CVD age-dependently into account. We estimated the RR based on values in the base case and the results of a meta-analysis among 147 randomized controlled trials [4].

We applied a CVD prediction model that estimated CVD risk in eight cumulative years. Therefore, a conservative ten-year time horizon was applied to examine the cost-effectiveness of the intervention in the base case. In addition, we ran a lifetime-horizon model to capture long-term effects of the screening and treatment.

Incremental cost-effectiveness ratios for each scenario were investigated from the health care provider perspective. The threshold for willingness to pay was three times the gross domestic product (GDP) [40], taking the PPP exchange rate into account [41]. One GDP per capita which is based on PPP in Vietnam in 2013 was 5,294 current Int$ [42].

### Ethical statement

The study on costs of stroke and MI was approved by the Ethical Committee of the Thai Nguyen General Hospital; the extraction of cost data was done with permission of the Planning Department of this hospital. The research proposal and consent procedure of the survey involving human participants was approved by the Institutional Review Board in Biomedical Research of the Institute of Social and Medical Studies in Vietnam. Participants were informed about the objectives and methods of the study and signed a consent form when they agreed to participate.

## Results

### Deterministic analysis

Results on the numbers of QALYs gained, incremental costs and cost-effectiveness ratios of the ten-year time horizon model are presented in Table 3. In comparison with no screening, all screening strategies resulted in QALYs gained. The number of QALYs gained from screening every two years was very similar to the number gained by an annual screening, in all corresponding age and sex groups. For example, QALYs gained in screening males from age 35 onwards was 0.25 and 0.26 in biannual and annual screening, respectively. Cost per QALYs gained varied by groups and screening strategies, from cost saving to Int$ 758,695 per QALY gained. Screening was not cost-effective for screening strategies starting at 35 years for both sexes in all strategies. Among females, one-off screening was cost-effective starting at 45 years at Int$ 12,070 per QALY gained. For screening starting at 55 years, one-off or biannual screening cost per QALY gained amounted to Int$ 871 and Int$ 11,189, respectively. Screening males from 55 years onwards, was cost saving, Int$ 2,076 and Int$ 7,638 for one-off, biannual and annual screening, respectively.

**Table 3:** Cost–effectiveness of screening for hypertension in alternative screening strategies for hypertension by age and sex in the 10 years model (per 1,000 people).

**Start screening at age of 35 years, female**					
No	47,676	8,942			
One-off	53,249	8,942	5,573	0.044	127,715
E1	94,728	8,942	47,052	0.062	758,695
E2	70,713	8,942	23,037	0.060	386,851
E1&T.20%	94,640	8,942	46,964	0.082	572,679
E2&T.20%	70,629	8,942	22,952	0.079	291,476
**Start screening at age of 45 years, female**					
No	143,627	8,421			
One-off	147,633	8,421	4,006	0.332	12,070
E1	181,734	8,422	38,107	0.361	105,525
E2	161,962	8,422	18,336	0.357	51,335
E1&T.20%	181,249	8,422	37,622	0.478	78,786
E2&T.20%	161,483	8,422	17,856	0.472	37,806
**Start screening at age of 55 years, female**					
No	231,456	7,278			
One-off	232,311	7,279	855	0.982	871
E1	257,492	7,279	26,036	1.022	25,471
E2	242,831	7,279	11,375	1.017	11,189
E1&T.20%	256,092	7,279	24,635	1.352	18,226
E2&T.20%	241,439	7,279	9,982	1.344	7,425
**Start screening at age of 35 years, male**					
No	99,013	8,436			
One-off	104,150	8,436	5,138	0.175	29,433
E1	140,394	8,436	41,381	0.262	158,147
E2	117,314	8,436	18,301	0.250	73,227
E1&T.20%	139,978	8,437	40,965	0.370	110,602
E2&T.20%	116,916	8,437	17,903	0.354	50,607
**Start screening at age of 45 years, male**					
No	174,638	7,681			
One-off	177,658	7,682	3,020	0.722	4,183
E1	206,873	7,682	32,235	0.858	37,580
E2	188,094	7,682	13,456	0.839	16,035
E1&T.20%	205,542	7,682	30,904	1.214	25,453
E2&T.20%	186,793	7,682	12,155	1.188	10,233
**Start screening at age of 55 years, male**					
No	274,570	5,954			
One-off	272,510	5,956	Dominant	1.920	Dominant
E1	290,298	5,956	15,728	2.059	7,638
E2	278,804	5,956	4,234	2.039	2,076
E1&T.20%	286,994	5,957	12,424	2.915	4,262
E2&T.20%	275,533	5,957	963	2.887	334

Note: No: No screening, One-off: screening once at the first year. E1: Annual screening, E2: Biannual screening, E1&T.20%: Annual screening combined with increasing coverage of treatment by 20%, E2&T.20%: Biannual screening combined with increasing coverage of treatment by 20%.

When we ran models for different starting ages (35 years, 45 years and 55 years) and sexes in comparison with no screening for the lifetime horizon, more QALYs were gained in all strategies. Especially combining screening with increasing treatment by 20% produced relatively high gains in QALYs, compared to only screening, in all ages and sexes. For example, this combination resulted in 4.88 vs 3.69 QALYs gained in the strategies of screening females from 35 years onwards. For the same age and screening strategy, QALYs gained in males were always higher than in females. For example, we saw 5.12 QALYs added for females versus 6.27 for males with annual screening starting at 45 years. The exact numbers of QALYs gained for each strategy is presented in S1 Fig.

**Fig 2:**
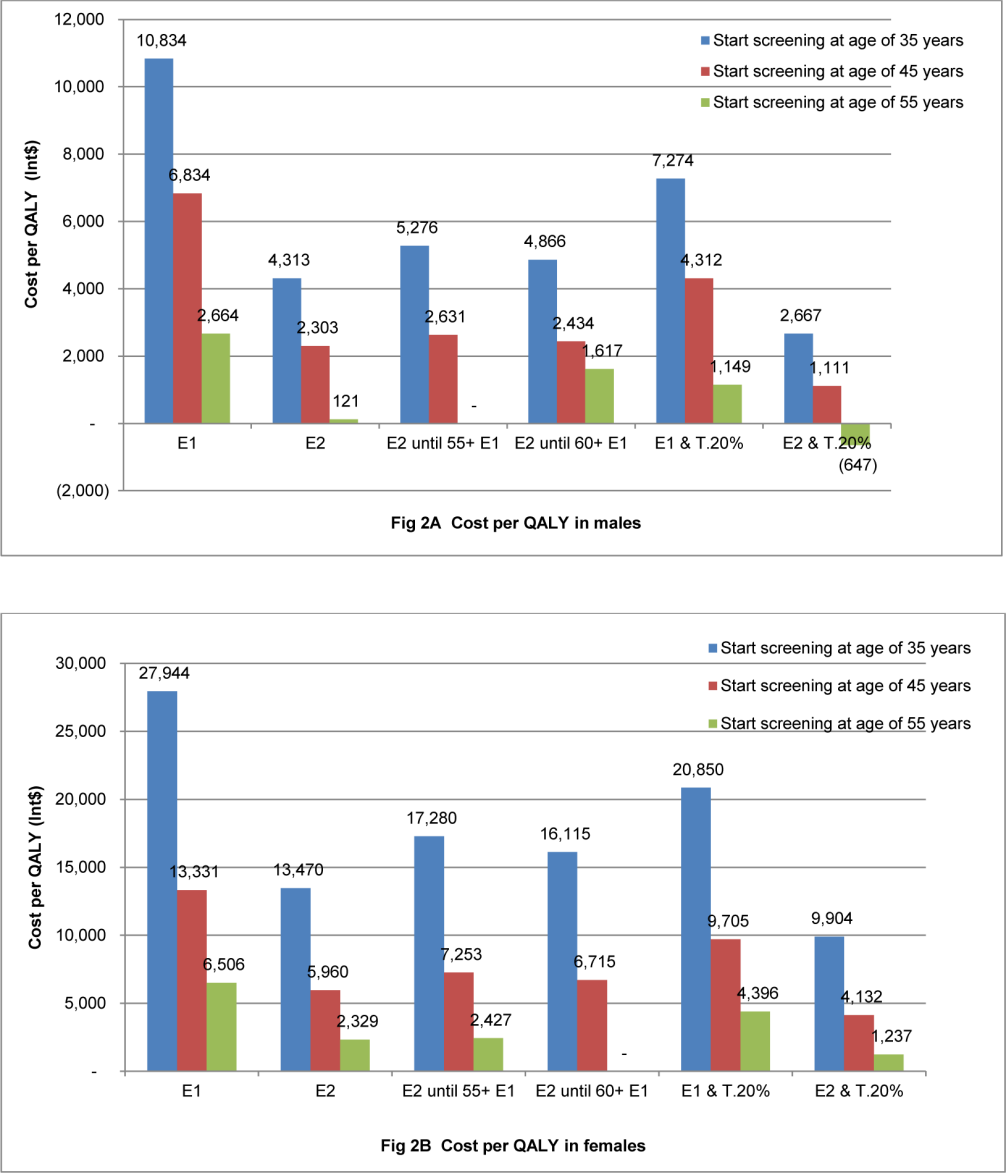
Cost per QALY by different strategies and age group, lifetime model. E1: Annual screening, E2: Biannual screening, E2 until 60+ E1: Biannual screening until 60 years old then annual screening until died, E2 until 55+ E1: Biannual screening until 55 years old then annual screening until died, E1&T.20%: Annual screening combined with increasing coverage of treatment by 20%, E2&T.20%: Biannual screening combined with increasing coverage of treatment by 20%.

Cost per QALY gained was less than the threshold of Int$ 15,883 per QALY in all scenarios, except for females from 35 years onwards. For example, cost per QALY in females from 35 years onwards were Int$ 27,944, Int$ 20,850, Int$ 17,280 and Int$ 16,115 in strategies of annual, annual combined with increased treatment by 20% and biannual up to 55 or 60 years and then annual screening, respectively. Cost per QALY gained in males was always lower than in females for the same age group and screening strategy. For example, in the annual strategy starting at 45 years, for males the cost was Int$ 6,834 per QALY gained, compared to Int$ 13,331 per QALY for females. Costs per QALY gained are shown in Fig 2.

### Probabilistic analysis

Full probabilistic sensitivity analysis of cost-effectiveness for a ten-year horizon is presented in S2 Fig. In the scenario of screening starting at 45 years, we found a 65% probability of being cost-effective in one-off screening in females and 95% in males. For the screening strategy in females starting at 55 years, the probability of favourable cost-effectiveness was 90% with one-off screening. For screening males starting at 55 years, all strategies gave a high probability of being cost-effective.

**Fig 3:**
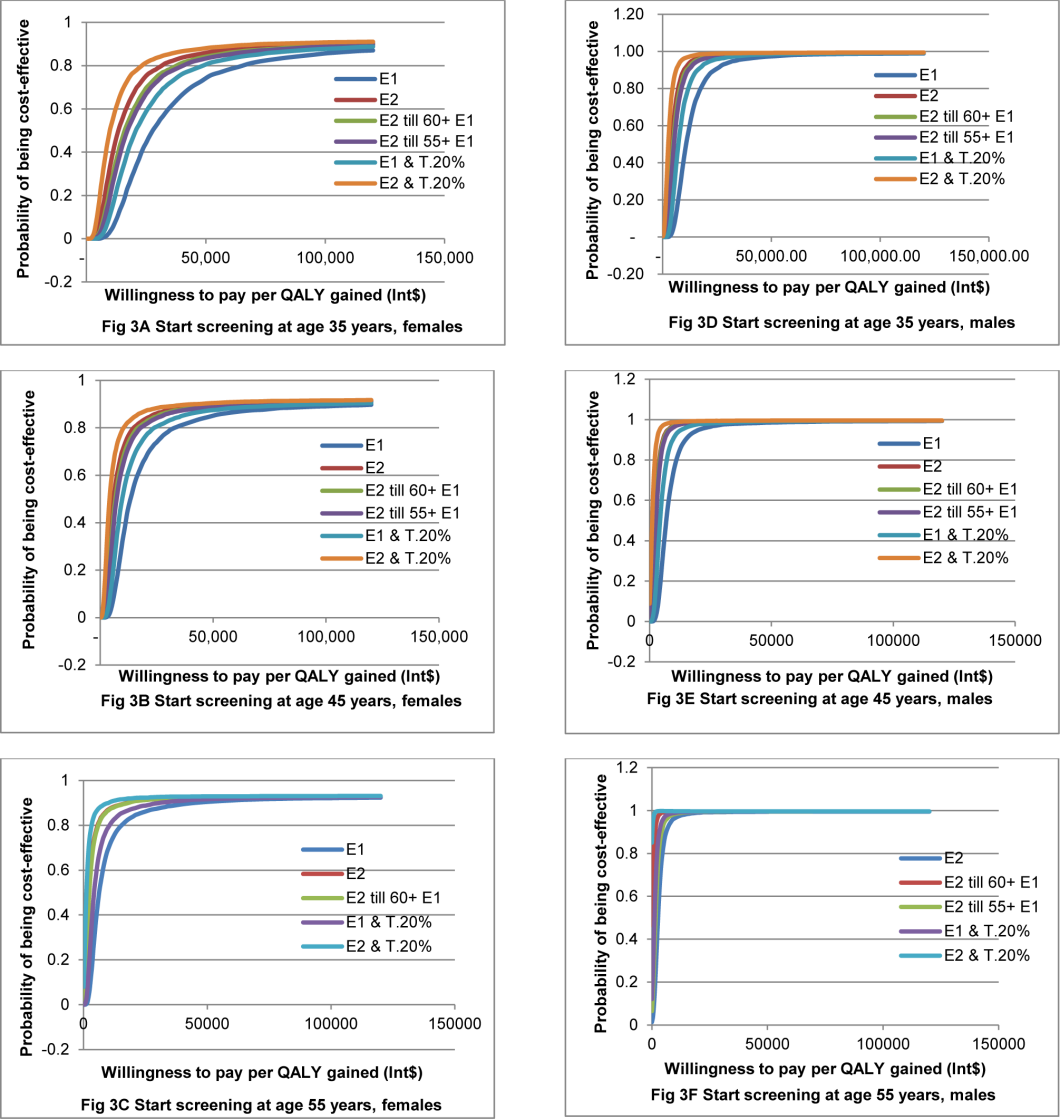
Cost-effectiveness acceptability curves of different screening strategies, lifetime horizon model. E1: Annual screening, E2: Biannual screening, E2 till 60+ E1: Biannual screening until 60 years old then annual screening until died, E2 till 55+ E1: Biannual screening until 55 years old then annual screening until died, E1&T.20%: Annual screening combined with increasing coverage of treatment by 20%, E2&T.20%: Biannual screening combined with increasing coverage of treatment by 20%.

The probability of favourable cost-effectiveness for each scenario using the lifetime horizon is shown in Fig 3. The strategy of screening repeated every two years combined with increasing treatment by 20% always gave the highest probability of being cost-effective, followed by biannual screening. Annual screening gave the lowest probability of being cost-effective in all scenarios.

### Univariate sensitivity analysis

To evaluate how the cost per QALY gained is affected by the variation in input variables, we conducted univariate sensitivity analysis in both time-horizon models. Results of cost per QALY gained in sensitivity analysis are presented in S2 Table for the ten-year time horizon and in S3 Table for lifetime. In the ten-year model, the results seem relatively insensitive to changing values of input parameters, except for a few cases. One example for the latter is decreased utilities of CVD by 20% for annual screening in females 55 years, where the result was lower than the threshold value, while in the base case it was higher than the threshold in the scenarios of annual screening alone or combined with 20% increased treatment. Also, in one-off screening, applying HBP prevalence of the national survey in males from 35 years onwards, the result became lower than the threshold, while it was higher than the threshold in the base case. In the scenario of applying utilities based on GBD, cost per QALY was much lower than in the base case but had similar trends in comparison with the threshold.

In the lifetime model, the cost per QALY gained was quite insensitive to changing values of input parameters in all different screening options, age and sex groups, with the exception of applying a 3% discount rate on utility and applying HBP prevalence by the national survey. Cost per QALY was much lower after changing the HBP prevalence and higher in a 3% utility discount scenario in comparison with the base case. Taking the threshold into account, results contrasted with the base case for screening females starting at 35 years in a sensitivity analysis of 25% decreased screening costs, 10% or 20% decreased CVD utility, or applying HBP prevalence from the national survey. Notably, the cost per QALY gained in these analyses changed from over to below the threshold in strategies of every two years to 55 or 60 years and then annual screening, or annual screening combined with 20% increased treatment. Screening was no longer cost-effective for annual screening of females starting at 45 years if increasing screening costs by 25%, reducing transition probability from hypertension to CVD by 25%, or discounting utilities.

## Discussion

This is one of the few studies on health economics analysis of screening for hypertension. It was conducted in the context of the current plans of the Ministry of Health in Vietnam to implement screening for hypertension as part of routine medical examinations to be covered by health insurance. However, in the context of limited resources, it may be useful to consider starting screening at a different age than that recommended by WHO. There is an urgent need for evidence to inform the new policies and planning, which should be based on local data to identify locally appropriate strategies, such as target age groups. Screening for hypertension starting at 35-, 45- or 55 years in males and at 55 years in females until death was cost-effective. The best screening strategies were biannual screening alone or combined with 20% increased treatment coverage. Also highly probable to be cost-effective was bi-annual screening of females starting at 45 years until death, either alone or combined with 20% increased treatment.

We found a negligible probability of favourable cost-effectiveness for screening females starting at 35 years using different strategies. These results may be explained by the lower HBP prevalence in this group compared to others. Overall, cost per QALY gained was lower than the threshold and showed a relatively high probability of being cost-effective in various screening strategies.

Regarding the initial age at screening, cost per QALY gained was lower in the older group in all strategies. This finding is consistent with a study in the Netherlands [9]. Results on cost-effectiveness of screening for hypertension and anti-hypertensive treatment in preventing CVD and kidney disease were also in line with our results [8].

Even though we found that various screening strategies could be cost-effective, biannual screening combined with increased treatment by 20% always had the highest probability of being cost-effective, among different strategies for the same age and sex. This finding agrees with previous studies demonstrating higher QALY gains with treatment, compared to no treatment [43]. Ergo, combining screening with increased treatment among those diagnosed with hypertension arises as a strong recommendation from our analysis.

In the 10 years model, cost per QALY gained was much higher than in the lifetime model, comparing the same age, sex, and screening strategy. This result was similar to that from a previous study comparing one-year, three-year or five-year horizons on the costs and effects of consistently similar hypertension treatment: as the time horizon increases, cost per QALY decreases [44]. We found a high probability of being cost-effective only in the scenario of screening females starting at 55 years and males at 45 years, when screening is done one-off or biannually combined with 20% increased treatment. For the screening strategy in males starting at 55 years, all strategies were cost-effective. This finding contributes sufficient evidence to recommend continuous screening and long-term treatment for HBP in these specific groups.

We applied coverage of treatment among patients who are aware of their diagnosis, using current treatment levels extracted from the national survey. This prevalence was estimated at a cut-off point taking adherence into account, at the time of the survey. We also ran the models with the assumption that all treated patients strictly comply with therapy. Previous studies in Vietnam found that among patients managed at CHS, patients’ adherence with anti-hypertension medicine (at cut-off point 80%) was 57% and 46% among females and males, respectively [45]. A similar study in China showed that the incremental cost-effectiveness ratio was sensitive to adherence levels. However, it was still lower than the threshold of three times the GDP, applying adherence levels of 75% or even down to 40% [46].

We built the models to estimate cost-effectiveness of screening and treatment for hypertension in a context of limited data. We were conservative in selecting values for input parameters and model to predict CVD of hypertensive patients. Yet, the first limitation to be noted does concern the input parameters which may not always exactly represent the Vietnamese population. The limited availability of data on health utilities should also be noted. Health utilities of acute strokes, acute and stable MI were extracted from data available from other countries and these were used for the Vietnamese context.

Several types of data/sources of information were not available in Vietnam. For example, there was no model to predict CVD risk or relative risk of treatment versus no treatment for hypertension or CVD. However, we had previously shown that an existing Asian model may be appropriate to predict CVD in Vietnam, as used in this study [14]. We also applied the results from a meta-analysis among Asian studies, which seems reasonable to weight the different CVD components and estimate the RR of treatment for HBP [3]. It should be pointed out that the RR reduction of CVD reported in the base case is not based on age-specific BP at pre-treatment. To examine uncertainty introduced here, one specific scenario was analyzed. The result of the scenario where the RR reduction of CVD depended on age showed that there were only small changes in the cost per QALY compared with the base case and the same trend remained when compared with the threshold.

Data on individual profiles (age, sex, BP, cholesterol level, smoking status) for hypertensive patients were not available. To overcome this limitation, we conducted a survey including almost 4,000 subjects, which provided the prevalence of hypertension and profile data of hypertensive patients as inputs to the model [14]. However, that study did not include urban regions. We examined the potential effects of this parameter by applying HBP prevalence from the national survey, which included both rural and urban areas, in a separate scenario.

We considered parameter estimates for the non-treatment group as adequately reflected by the less intensive treatment or placebo/reference group in a meta-analysis among Asian populations [3]. The relative risk of treatment and non-treatment groups that we extracted from that study may be considered appropriate for our purposes, despite potential adherence issues.

With regard to adherence, in a report from China the estimated adherence was 40% in both sexes (50% continuation of prescribed medications, with 10% of doses missed among patients continuing treatment). In a previous study in Vietnam [45] we estimated adherence among patients whose HBP was managed at CHS; patients managed at other health facilities were not included. When new data becomes available in future, we can update the model to explore how the cost-effectiveness ratio changes if the level of adherence changes. In general, limitations in available data meant we could not validate the model as extensively as recommended [47].

## Conclusion

From a health economics perspective, integrating screening for hypertension into routine medical examination and related coverage by health insurance could be recommended. Our current model suggests that screening for hypertension provides a high probability of being cost-effective in preventing CVD. A screening strategy should be selected based on age, sex and screening interval. Screening of males starting at 35-, 45-, or 55 years and females at 55 years until death displayed high probabilities of being cost-effective in all strategies. Screening in females starting at 45 years displayed a high probability of being cost-effective for biannual screening alone or combined with 20% increased treatment. Screening females starting at 35 years gave a low probability of being cost-effective in all strategies, except for biannual screening with increased treatment which still had a 70% probability of being cost-effective. Consistently, lower costs per QALY were found for males than for females. The lifetime model provided lower cost per QALY gained than the 10-year model. Our results may inform and help managers and policy-makers in developing guidelines for hypertension management in Vietnam.

## Supporting Information

S1 Dataset (XLSX)

S1 Table Description of screening strategies. (DOCX)

S2 Table Results of univariate sensitivity analysis in 10 years horizon model. (DOCX)

S3 Table Results of univariate sensitivity analysis in lifetime horizon model. (DOCX)

S1 Fig QALYs gained by different screening strategies, lifetime model. (TIF)

S2 Fig Cost-effectiveness acceptability curves of different screening strategies, 10 years horizon model. (TIF)
