# Supplementary material for: Cost-Effectiveness Analysis of Screening for and Managing Identified Hypertension for Cardiovascular Disease Prevention in Vietnam
Source: PLoS One. 2016 May 18;11(5):e0155699. doi: 10.1371/journal.pone.0155699 (PMC4871542; doi:10.1371/journal.pone.0155699)
Supplement: S1 Table — (DOCX) [file pone.0155699.s002.docx]

**S1Table: Description of screening strategies**

| **Ten-years horizon** | **Screening scenario** | **Description** |
| --- | --- | --- |
| Male, 35 years old | One-off screening | Cohort of 35 years old. Screening once in the first year. Follow up until they are 45 years old or died. |
|  | Annual screening | Cohort of 35 years old. Start screening at age 35 years old and repeat every year until 45 years old or died. |
|  | Biannual screening | Cohort of 35 years old. Start screening at age 35 years old and repeat every two years until 45 years old or died. |
|  | Annual screening and increase coverage of treatment by 20% | Cohort of 35 years old. Start screening at age 35 years old and repeat every year until 45 years old or die. And increase coverage of treatment by 20%. |
|  | Biannual screening and increase coverage of treatment by 20% | Cohort of 35 years old. Start screening at age 35 years old and repeat every two years until 45 years old or died. And increase coverage of treatment by 20%. |
| Male, 45 years old | One-off screening | Cohort of 45 years old. Screening once in the first year. Follow up until they are 55 years old or died. |
|  | Annual screening | Cohort of 45 years old. Start screening at age 45 years old and repeat every year until 55 years old or died. |
|  | Biannual screening | Cohort of 45 years old. Start screening at age 45 years old and repeat every two years until 55 years old or died. |
|  | Annual screening and increase coverage of treatment by 20% | Cohort of 45 years old. Start screening at age 45 years old and repeat every year until 55 years old or died. And increase coverage of treatment by 20%. |
|  | Biannual screening and increase coverage of treatment by 20% | Cohort of 45 years old. Start screening at age45 years old and repeat every two years until 55 years old or died. And increase coverage of treatment by 20%. |
| Male, 55 years old | One-off screening | Cohort of 55 years old. Screening once in the first year. Follow up until they are 65 years or died. |
|  | Annual screening | Cohort of 55 years old. Start screening at age 55 years old and repeat every year until 65 years old or died. |
|  | Biannual screening | Cohort of 55 years old. Start screening at age 55 years old and repeat every two years until 65 years old or died. |
|  | Annual screening and increase coverage of treatment by 20% | Cohort of 55 years old. Start screening at age 55 years old and repeat every year until 65 years old or died. And increase coverage of treatment by 20% |
|  | Biannual screening and increase coverage of treatment by 20% | Cohort of 55 years old. Start screening at age55 years old and repeat every two years until 65 years old or died. And increase coverage of treatment by 20% |
| Female, 35 years old | One-off screening | Cohort of 35 years old. Screening once in the first year. Follow up until they are 45 years or died. |
|  | Annual screening | Cohort of 35 years old. Start screening at age 35 years old and repeat every year until 45 years old or died. |
|  | Biannual screening | Cohort of 35 years old. Start screening at age 35 years old and repeat every two years until 45 years old or died. |
|  | Annual screening and increase coverage of treatment by 20% | Cohort of 35 years old. Start screening at age 35 years old and repeat every year until 45 years old or died. And increase coverage of treatment by 20%. |
|  | Biannual screening and increase coverage of treatment by 20% | Cohort of 35 years old. Start screening at age 35 years old and repeat every two years until 45 years old or died. And increase coverage of treatment by 20% |
| Female, 45 years old | One-off screening | Cohort of 45 years old. Screening once in the first year. Follow up until they are 55 years or died. |
|  | Annual screening | Cohort of 45 years old. Start screening at age 45 years old and repeat every year until 55 years old or died. |
|  | Biannual screening | Cohort of 45 years old. Start screening at age 45 years old and repeat every two years until 55 years old or died. |
|  | Annual screening and increase coverage of treatment by 20% | Cohort of 45 years old. Start screening at age 45 years old and repeat every year until 55 years old or died. And increase coverage of treatment by 20% |
|  | Biannual screening and increase coverage of treatment by 20% | Cohort of 45 years old. Start screening at age 45 years old and repeat every two years until 55 years old or died. And increase coverage of treatment by 20% |
| Female, 55 years old | One-off screening | Cohort of 55 years old. Screening once in the first year. Follow up until they are 65 years or died. |
|  | Annual screening | Cohort of 55 years old. Start screening at age 55 years old and repeat every year until 65 years old or died. |
|  | Biannual screening | Cohort of 55 years old. Start screening at age 55 years old and repeat every two years until 65 years old or died. |
|  | Annual screening and increase coverage of treatment by 20% | Cohort of 55 years old. Start screening at age 55 years old and repeat every year until 65 years old or died. And increase coverage of treatment by 20%. |
|  | Biannual screening and increase coverage of treatment by 20% | Cohort of 55 years old. Start screening at age55 years old and repeat every two years until 65 years old or died. And increase coverage of treatment by 20%. |
| **Lifetime horizon** | **Screening scenario** | **Description** |
| Female, 35 years old | Annual screening | Cohort of 35 years old. Start screening at age 35 years old and repeat every year until 82 years old or died (47 years follow up). |
|  | Biannual screening | Cohort of 35 years old. Start screening at age 35 years old and repeat every two years until 82 years old or died (47 years follow up). |
|  | Biannual screening until 55 years, then annual screening | Cohort of 35 years old. Start screening at age 35 years old and repeat every two years until 55 years old or died. Then repeat screening every year, from 56 to 82 years old or die (47 years follow up). |
|  | Biannual screening until 60 years, then annual screening | Cohort of 35 years old. Start screening at age 35 years old and repeat every two years until 60 years old or died. Then, repeat screening every year, from 61 to 82 years old or died (47 years follow up). |
|  | Annual screening and increase coverage of treatment by 20% | Cohort of 35 years old. Start screening at age 35 years old and repeat every year until 82 years old or died (47 years follow up). And increase coverage of treatment by 20%. |
|  | Biannual screening and increase coverage of treatment by 20% | Cohort of 35 years old. Start screening at age 35 years old and repeat every two years until 82 years old or died (47 years follow up). And increase coverage of treatment by 20%. |
| Female, 45 years old | Annual screening | Cohort of 45 years old. Start screening at age 45 years old and repeat every year until 83 years old or died (38 years follow up). |
|  | Biannual screening | Cohort of 45 years old. Start screening at age 45 years old and repeat every two years until 83 years old or died (38 years follow up). |
|  | Biannual screening until 55 years, then annual screening | Cohort of 45 years old. Start screening at age 45 years old and repeat every two years until 55 years old or died. Then, repeat screening every year, from 56 to 83 years old or died (38 years follow up). |
|  | Biannual screening until 60 years, then annual screening | Cohort of 45 years old. Start screening at age 45 years old and repeat every two years until 60 years old or died. Then, repeat screening every year, from 61 to 83 years old or died (38 years follow up). |
|  | Annual screening and increase coverage of treatment by 20% | Cohort of 45 years old. Start screening at age 45 years old and repeat every year until 83 years old or died (38 years follow up). And increase coverage of treatment by 20%. |
|  | Biannual screening and increase coverage of treatment by 20% | Cohort of 45 years old. Start screening at age 45 years old and repeat every two years until 83 years old or died (38 years follow up). And increase coverage of treatment by 20%. |
| Female, 55 years old | Annual screening | Cohort of 55 years old. Screening in every year, starting from 55 to 84 years old or died (29 years follow up). |
|  | Biannual screening | Cohort of 55 years old. Screening in every two years, starting from 55 to 84 years old or died (29 years follow up). |
|  | Annual screening and increase coverage of treatment by 20% | Cohort of 55 years old. Screening in every year, starting from 55 to 84 years old or died (29 years follow up). And increase coverage of treatment by 20% . |
|  | Biannual screening and increase coverage of treatment by 20% | Cohort of 55 years old. Screening in every two years, starting from 55 to 84 years old or died (29 years follow up). And increase coverage of treatment by 20%. |
| Male, 35 years old | Annual screening | Cohort of 35 years old. Start screening at age 35 years old and repeat every year until 74 years old or died (39 years follow up). |
|  | Biannual screening | Cohort of 35 years old. Start screening at age 35 years old and repeat every two years until 74 years old or died (39 years follow up). |
|  | Biannual screening until 55 years, then annual screening | Cohort of 35 years old. Start screening at age 35 years old and repeat every two years until 55 years old or died. Then repeat screening every years, from 56 to 74 years old or died (39 years follow up). |
|  | Biannual screening until 60 years, then annual screening | Cohort of 35 years old. Start screening at age 35 years old and repeat every two years until 60 years old or died. Then, repeat screening every year, from 61 to 74 years old or died (39 years follow up). |
|  | Annual screening and increase coverage of treatment by 20% | Cohort of 35 years old. Start screening at age 35 years old and repeat every year until 74 years old or died (39 years follow up). And increase coverage of treatment by 20%. |
|  | Biannual screening and increase coverage of treatment by 20% | Cohort of 35 years old. Start screening at age 35 years old and repeat every two years until 74 years old or died (39 years follow up). And increase coverage of treatment by 20%. |
| Male, 45 years old | Annual screening | Cohort of 45 years old. Start screening at age 45 years old and repeat every year until 76 years old or died (31 years follow up). |
|  | Biannual screening | Cohort of 45 years old. Start screening at age 45 years old and repeat every two years until 76 years old or died (31 years follow up) |
|  | Biannual screening until 55 years, then annual screening | Cohort of 45 years old. Start screening at age 45 years old and repeat every two years until 55 years old. Then, repeat screening every year, from 56 to 76 years old or died (31 years follow up) |
|  | Biannual screening until 60 years, then annual screening | Cohort of 45 years old. Start screening at age 45 years old and repeat every two years until 60 years old. Then, repeat screening every year, from 61 to 76 years old or died (31 years follow up) |
|  | Annual screening and increase coverage of treatment by 20% | Cohort of 45 years old. Start screening at age 45 years old and repeat every year until 76 years old or died (31 years follow up). And increase coverage of treatment by 20%. |
|  | Biannual screening and increase coverage of treatment by 20% | Cohort of 45 years old. Start screening at age 45 years old and repeat every two years until 76 years old or died (31 years follow up). And increase coverage of treatment by 20%. |
| Male, 55 years old | Annual screening | Cohort of 55 years old. Screening in every year, starting from 55 to 77 years old or died (22 years follow up) |
|  | Biannual screening | Cohort of 55 years old. Screening in every two years, starting from 55 to 77 years old or died (22 years follow up) |
|  | Annual screening and increase coverage of treatment by 20% | Cohort of 55 years old. Start screening at age 55 years old and repeat every year until 77 years old or died (22 years follow up). And increase coverage of treatment by 20%. |
|  | Biannual screening and increase coverage of treatment by 20% | Cohort of 55 years old. Start screening at age 55 years old and repeat every two years until 77 years old or died (22 years follow up). And increase coverage of treatment by 20%. |
| **Notes**: 48%, 62% treatment among hypertension were applied in this study in male and female, respectively | | |
