# Supplementary material for: Cost-Effectiveness Analysis of Screening for and Managing Identified Hypertension for Cardiovascular Disease Prevention in Vietnam
Source: PLoS One. 2016 May 18;11(5):e0155699. doi: 10.1371/journal.pone.0155699 (PMC4871542; doi:10.1371/journal.pone.0155699)
Supplement: S2 Table — (DOCX) [file pone.0155699.s003.docx]

**S2 Table: Results of univariate sensitivity analysis in 10 years horizon model**

|  | Base case | (+) 25% screening cost | (-) 25% screening cost | (+) 25% hypertension treatment cost | (-) 25% hypertension treatment cost | (+) 25% CVD cost | (-) 25% CVD cost | (-) 10% CVD utility | (-) 20% CVD utility | (+) 25% transition probability from hypertension to CVD | (-) 25% transition probability from hypertension to CVD | 1% utility discount | 3% utility discount | RR reduction of CVD depend on age | Applying utilities from Global Burden Disease | hypertension prevalence by national survey |
| --- | --- | --- | --- | --- | --- | --- | --- | --- | --- | --- | --- | --- | --- | --- | --- | --- |
| **Start screening at age of 35 years, females** |  |  |  |  |  |  |  |  |  |  |  |  |  |  |  |  |
| One-off | 127,715 | 160,645 | 94,784 | 127,734 | 127,696 | 126,694 | 128,735 | 90,409 | 69,970 | 101,589 | 171,254 | 137,060 | 157,250 | 110,558 | 69,737 | 43,362 |
| E1 | 758,695 | 949,470 | 567,920 | 758,713 | 758,676 | 757,575 | 759,815 | 538,265 | 417,086 | 607,256 | 1,011,093 | 816,576 | 942,381 | 658,539 | 415,909 | 306,054 |
| E2 | 386,851 | 488,753 | 284,949 | 382,783 | 390,920 | 385,730 | 387,972 | 274,463 | 212,677 | 309,219 | 516,236 | 416,384 | 480,576 | 335,613 | 212,078 | 150,707 |
| E1 & T.20% | 572,679 | 716,950 | 428,408 | 572,698 | 572,661 | 571,560 | 573,799 | 406,294 | 314,825 | 458,154 | 763,554 | 616,370 | 711,330 | 496,994 | 313,937 | 230,418 |
| E2 & T.20% | 291,476 | 368,539 | 214,414 | 288,404 | 294,549 | 290,356 | 292,597 | 206,797 | 160,243 | 232,767 | 389,324 | 313,728 | 362,094 | 252,784 | 159,792 | 112,939 |
| **Start screening at age of 45 years, females** |  |  |  |  |  |  |  |  |  |  |  |  |  |  |  |  |
| One-off | 12,070 | 16,103 | 8,038 | 12,089 | 12,051 | 11,036 | 13,104 | 8,543 | 6,611 | 8,886 | 17,369 | 12,948 | 14,843 | 9,313 | 6,590 | 5,018 |
| E1 | 105,525 | 132,949 | 78,102 | 105,544 | 105,506 | 104,464 | 106,586 | 74,735 | 57,854 | 83,933 | 141,506 | 113,290 | 130,081 | 85,054 | 57,678 | 53,532 |
| E2 | 51,335 | 65,768 | 36,902 | 50,796 | 51,874 | 50,275 | 52,395 | 36,356 | 28,144 | 40,407 | 69,542 | 55,112 | 63,278 | 41,101 | 28,058 | 25,150 |
| E1 & T.20% | 78,786 | 99,525 | 58,048 | 78,805 | 78,767 | 77,725 | 79,847 | 55,798 | 43,194 | 62,455 | 105,999 | 84,584 | 97,119 | 63,368 | 43,063 | 39,476 |
| E2 & T.20% | 37,806 | 48,721 | 26,891 | 37,403 | `38,209 | 36,746 | 38,866 | 26,775 | 20,727 | 29,539 | 51,578 | 40,587 | 46,602 | 30,129 | 20,664 | 18,013 |
| **Start screening at age of 55 years, females** |  |  |  |  |  |  |  |  |  |  |  |  |  |  |  |  |
| One-off | 871 | 2,139 | Dominant | 890 | 852 | Dominant | 1,940 | 616 | 477 | Dominant | 2,530 | 933 | 1,067 | 466 | 476 | Dominant |
| E1 | 25,471 | 32,901 | 18,040 | 25,490 | 25,452 | 24,389 | 26,552 | 18,032 | 13,956 | 19,695 | 35,082 | 27,299 | 31,240 | 21,407 | 13,917 | 18,453 |
| E2 | 11,189 | 15,190 | 7,187 | 11,065 | 11,312 | 10,107 | 12,270 | 7,921 | 6,131 | 8,178 | 16,191 | 11,992 | 13,723 | 9,254 | 6,113 | 7,639 |
| E1 & T.20% | 18,226 | 23,845 | 12,607 | 18,245 | 18,207 | 17,144 | 19,307 | 12,903 | 9,987 | 13,852 | 25,500 | 19,534 | 22,354 | 15,242 | 9,959 | 12,921 |
| E2 & T.20% | 7,425 | 10,452 | 4,399 | 7,337 | 7,514 | 6,344 | 8,507 | 5,257 | 4,069 | 5,143 | 11,214 | 7,958 | 9,107 | 6,052 | 4,057 | 4,743 |
| **Start screening at age of 35 years, males** |  |  |  |  |  |  |  |  |  |  |  |  |  |  |  |  |
| One-off | 29,433 | 37,655 | 21,211 | 29,449 | 29,417 | 28,554 | 30,312 | 21,752 | 24,884 | 22,942 | 40,246 | 31,570 | 36,183 | 25,427 | 18,948 | 10,796 |
| E1 | 158,147 | 198,640 | 117,654 | 158,163 | 158,131 | 157,175 | 159,119 | 117,103 | 148,331 | 126,167 | 211,443 | 170,189 | 196,356 | 137,837 | 102,162 | 74,341 |
| E2 | 73,227 | 95,430 | 51,023 | 70,304 | 76,149 | 72,252 | 74,201 | 54,225 | 75,592 | 58,018 | 98,570 | 78,809 | 90,941 | 63,658 | 47,307 | 32,602 |
| E1 & T.20% | 110,602 | 139,209 | 81,995 | 110,618 | 110,586 | 109,630 | 111,574 | 81,898 | 111,963 | 88,007 | 148,257 | 119,024 | 137,324 | 96,306 | 71,448 | 51,446 |
| E2 & T.20% | 50,607 | 66,292 | 34,921 | 48,547 | 52,667 | 49,633 | 51,581 | 37,474 | 56,955 | 39,860 | 68,513 | 54,464 | 62,849 | 43,900 | 32,694 | 21,959 |
| **Start screening at age of 45 years, males** |  |  |  |  |  |  |  |  |  |  |  |  |  |  |  |  |
| One-off | 4,183 | 6,119 | 2,247 | 4,199 | 4,167 | 3,277 | 5,089 | 3,091 | 2,351 | 2,661 | 6,709 | 4,482 | 5,126 | 2,991 | 2,693 | 1,618 |
| E1 | 37,580 | 47,909 | 27,252 | 37,596 | 37,564 | 36,631 | 38,530 | 27,798 | 20,575 | 29,523 | 50,999 | 40,331 | 46,276 | 30,196 | 24,238 | 22,857 |
| E2 | 16,035 | 21,694 | 10,377 | 15,334 | 16,736 | 15,086 | 16,984 | 11,861 | 10,003 | 12,175 | 22,458 | 17,209 | 19,745 | 12,605 | 10,342 | 9,079 |
| E1 & T.20% | 25,453 | 32,750 | 18,157 | 25,469 | 25,437 | 24,504 | 26,403 | 18,828 | 15,362 | 19,756 | 34,939 | 27,317 | 31,343 | 20,297 | 16,417 | 15,068 |
| E2 & T.20% | 10,233 | 14,230 | 6,235 | 9,742 | 10,723 | 9,284 | 11,182 | 7,569 | 7,367 | 7,500 | 14,775 | 10,982 | 12,600 | 7,870 | 6,600 | 5,335 |
| **Start screening at age of 55 years, males** |  |  |  |  |  |  |  |  |  |  |  |  |  |  |  |  |
| One-off | Dominant | Dominant | Dominant | Dominant | Dominant | Dominant | Dominant | Dominant | 170 | Dominant | Dominant | Dominant | Dominant | Dominant | Dominant | Dominant |
| E1 | 7,638 | 10,513 | 4,763 | 7,654 | 7,622 | 6,657 | 8,619 | 5,647 | 4,964 | 5,426 | 11,302 | 8,170 | 9,311 | 6,254 | 4,925 | 5,928 |
| E2 | 2,076 | 3,724 | 428 | 1,928 | 2,224 | 1,095 | 3,057 | 1,535 | 2,179 | 918 | 3,983 | 2,221 | 2,531 | 1,510 | 1,339 | 1,253 |
| E1 & T.20% | 4,262 | 6,293 | 2,232 | 4,279 | 4,246 | 3,281 | 5,244 | 3,152 | 3,552 | 2,689 | 6,862 | 4,559 | 5,196 | 3,374 | 2,748 | 3,057 |
| E2 & T.20% | 334 | 1,498 | Dominant | 234 | 433 | Dominant | 1,315 | 247 | 1,446 | Dominant | 1,691 | 357 | 407 | 23 | 215 | Dominant |

*Note: One-off: screening once in the first year, E1: Annual screening, E2: Biannual screening, E1&T.20%: Annual screening combined with increasing coverage of treatment by 20%, E2&T.20%: Biannual screening combined with increasing coverage of treatment by 20%, CVD: cardiovascuslar disease.
