# Supplementary material for: Cost-Effectiveness Analysis of Screening for and Managing Identified Hypertension for Cardiovascular Disease Prevention in Vietnam
Source: PLoS One. 2016 May 18;11(5):e0155699. doi: 10.1371/journal.pone.0155699 (PMC4871542; doi:10.1371/journal.pone.0155699)
Supplement: S3 Table — (DOCX) [file pone.0155699.s004.docx]

**S3 Table: Results of univariate sensitivity analysis in lifetime horizon model**

|  | Base case | (+) 25% screening cost | (-) 25% screening cost | (+) 25% hypertension treatment cost | (-) 25% hypertension treatment cost | (+) 25% CVD treatment cost | (-) 25% CVD treatment cost | (-) 10% CVD utility | (-) 20% CVD utility | (+) 25% transition probability from hypertension to CVD | (-) 25% transition probability from hypertension to CVD | 1% utility discount | 3% utility discount | RR reduction of CVD depend on age | Applying utilities from Global Burden Disease | Hypertension prevalence by national survey |
| --- | --- | --- | --- | --- | --- | --- | --- | --- | --- | --- | --- | --- | --- | --- | --- | --- |
| **Start screening at age of 35 years, females** |  |  |  |  |  |  |  |  |  |  |  |  |  |  |  |  |
| E1 | 27,944 | 35,220 | 20,669 | 27,954 | 27,935 | 27,646 | 28,243 | 19,510 | 14,987 | 22,899 | 36,366 | 38,728 | 71,742 | 28,587 | 14,938 | 14,639 |
| E2 | 13,470 | 17,277 | 9,664 | 13,330 | 13,610 | 13,171 | 13,769 | 9,405 | 7,225 | 10,924 | 17,712 | 18,678 | 34,639 | 13,849 | 7,201 | 6,654 |
| E2 until 55+ E1 | 17,280 | 22,000 | 12,560 | 17,179 | 17,381 | 16,981 | 17,579 | 12,065 | 9,268 | 14,075 | 22,623 | 23,967 | 44,478 | 17,749 | 9,238 | 8,759 |
| E2 until 60+ E1 | 16,115 | 20,556 | 11,675 | 16,003 | 16,228 | 15,816 | 16,414 | 11,252 | 8,644 | 13,111 | 21,122 | 22,353 | 41,483 | 16,571 | 8,616 | 8,116 |
| E1&T.20% | 20,850 | 26,352 | 15,348 | 20,860 | 20,841 | 20,552 | 21,149 | 14,558 | 11,183 | 17,028 | 27,226 | 28,896 | 53,529 | 21,353 | 11,146 | 10,792 |
| E2&T.20% | 9,904 | 12,783 | 7,026 | 9,801 | 10,008 | 9,605 | 10,203 | 6,915 | 5,312 | 7,971 | 13,119 | 13,733 | 25,469 | 10,207 | 5,295 | 4,754 |
| **Start screening at age of 45 years, females** |  |  |  |  |  |  |  |  |  |  |  |  |  |  |  |  |
| E1 | 13,331 | 17,049 | 9,612 | 13,343 | 13,319 | 12,933 | 13,728 | 9,305 | 7,147 | 10,748 | 17,624 | 16,916 | 26,480 | 13,855 | 7,125 | 6,847 |
| E2 | 5,960 | 7,910 | 4,011 | 5,898 | 6,022 | 5,563 | 6,358 | 4,161 | 3,196 | 4,639 | 8,144 | 7,564 | 11,842 | 6,271 | 3,186 | 2,682 |
| E2 until 55+ E1 | 7,253 | 9,514 | 4,993 | 7,204 | 7,303 | 6,855 | 7,651 | 5,063 | 3,889 | 5,710 | 9,807 | 9,206 | 14,419 | 7,611 | 3,877 | 3,412 |
| E2 until 60+ E1 | 6,715 | 8,846 | 4,584 | 6,660 | 6,770 | 6,317 | 7,113 | 4,687 | 3,600 | 5,264 | 9,115 | 8,523 | 13,347 | 7,056 | 3,589 | 3,108 |
| E1&T.20% | 9,705 | 12,517 | 6,893 | 9,717 | 9,693 | 9,308 | 10,103 | 6,775 | 5,203 | 7,742 | 12,962 | 12,316 | 19,278 | 10,123 | 5,187 | 4,806 |
| E2&T.20% | 4,132 | 5,606 | 2,657 | 4,088 | 4,175 | 3,734 | 4,529 | 2,884 | 2,215 | 3,122 | 5,793 | 5,243 | 8,209 | 4,387 | 2,208 | 1,655 |
| **Start screening at age of 55 years, females** |  |  |  |  |  |  |  |  |  |  |  |  |  |  |  |  |
| E1 | 6,506 | 8,670 | 4,342 | 6,521 | 6,492 | 5,954 | 7,058 | 4,548 | 3,496 | 4,978 | 9,023 | 7,704 | 10,606 | 7,991 | 3,486 | 4,518 |
| E2 | 2,329 | 3,490 | 1,168 | 2,303 | 2,355 | 1,777 | 2,881 | 1,628 | 1,251 | 1,510 | 3,659 | 2,758 | 3,797 | 3,103 | 1,248 | 1,314 |
| E2 until 60+ E1 | 2,427 | 3,612 | 1,243 | 2,402 | 2,453 | 1,875 | 2,980 | 1,697 | 1,304 | 1,591 | 3,786 | 2,875 | 3,957 | 3,220 | 1,301 | 1,390 |
| E1&T.20% | 4,396 | 6,032 | 2,759 | 4,410 | 4,381 | 3,843 | 4,948 | 3,073 | 2,362 | 3,225 | 6,314 | 5,205 | 7,166 | 5,521 | 2,355 | 2,894 |
| E2&T.20% | 1,237 | 2,115 | 359 | 1,221 | 1,253 | 685 | 1,789 | 865 | 665 | 603 | 2,257 | 1,465 | 2,016 | 1,825 | 663 | 471 |
| **Start screening at age of 35 years, males** |  |  |  |  |  |  |  |  |  |  |  |  |  |  |  |  |
| E1 | 10,834 | 13,865 | 7,802 | 10,843 | 10,824 | 10,501 | 11,167 | 7,926 | 6,249 | 8,703 | 14,376 | 14,050 | 22,974 | 9,468 | 6,873 | 6,237 |
| E2 | 4,313 | 5,926 | 2,701 | 4,112 | 4,514 | 3,980 | 4,646 | 3,156 | 2,488 | 3,313 | 5,964 | 5,597 | 9,165 | 3,736 | 2,736 | 2,137 |
| E2 until 55+ E1 | 5,276 | 7,098 | 3,453 | 5,106 | 5,446 | 4,942 | 5,609 | 3,860 | 3,043 | 4,108 | 7,207 | 6,849 | 11,221 | 4,584 | 3,347 | 2,742 |
| E2 until 60+ E1 | 4,866 | 6,599 | 3,132 | 4,682 | 5,049 | 4,532 | 5,199 | 3,560 | 2,807 | 3,769 | 6,677 | 6,316 | 10,347 | 4,225 | 3,087 | 2,484 |
| E1&T.20% | 7,274 | 9,416 | 5,132 | 7,283 | 7,264 | 6,941 | 7,607 | 5,322 | 4,196 | 5,759 | 12,737 | 9,434 | 15,425 | 6,337 | 4,614 | 4,032 |
| E2&T.20% | 2,667 | 3,806 | 1,528 | 2,528 | 2,806 | 2,334 | 3,000 | 1,951 | 1,538 | 1,951 | 3,842 | 3,461 | 5,667 | 2,287 | 1,692 | 1,136 |
| **Start screening at age of 45 years, males** |  |  |  |  |  |  |  |  |  |  |  |  |  |  |  |  |
| E1 | 6,834 | 8,982 | 4,686 | 6,846 | 6,823 | 6,383 | 7,285 | 5,002 | 3,945 | 5,322 | 9,332 | 8,252 | 11,784 | 6,088 | 4,340 | 4,204 |
| E2 | 2,303 | 3,464 | 1,142 | 2,169 | 2,437 | 1,852 | 2,755 | 1,686 | 1,329 | 1,565 | 3,506 | 2,781 | 3,974 | 2,018 | 1,463 | 1,067 |
| E2 until 55+ E1 | 2,631 | 3,865 | 1,398 | 2,508 | 2,755 | 2,180 | 3,083 | 1,926 | 1,519 | 1,836 | 3,929 | 3,178 | 4,542 | 2,315 | 1,671 | 1,294 |
| E2 until 60+ E1 | 2,434 | 3,624 | 1,244 | 2,304 | 2,563 | 1,982 | 2,885 | 1,781 | 1,405 | 1,673 | 3,674 | 2,939 | 4,200 | 2,137 | 1,546 | 1,157 |
| E1&T.20% | 4,312 | 5,830 | 2,794 | 4,324 | 4,300 | 3,861 | 4,763 | 3,156 | 2,489 | 3,229 | 6,091 | 5,206 | 7,435 | 3,821 | 2,738 | 2,459 |
| E2&T.20% | 1,111 | 1,931 | 290 | 1,019 | 1,202 | 659 | 1,562 | 813 | 641 | 575 | 1,974 | 1,341 | 1,916 | 946 | 705 | 242 |
| **Start screening at age of 55 years, males** |  |  |  |  |  |  |  |  |  |  |  |  |  |  |  |  |
| E1 | 2,664 | 3,954 | 1,374 | 2,678 | 2,650 | 2,026 | 3,302 | 1,954 | 1,542 | 1,719 | 4,197 | 3,020 | 3,835 | 2,667 | 1,698 | 1,925 |
| E2 | 121 | 849 | Dominant | 62 | 181 | Dominant | 759 | 89 | 70 | Dominant | 931 | 137 | 174 | 173 | 77 | Dominant |
| E2 until 60+ E1 | 1,617 | 2,676 | 558 | 1,600 | 1,634 | 979 | 2,255 | 1,186 | 936 | 850 | 2,853 | 1,834 | 2,329 | 1,643 | 1,031 | 1,036 |
| E1&T.20% | 1,149 | 2,061 | 238 | 1,163 | 1,136 | 512 | 1,787 | 843 | 666 | Dominant | 2,253 | 1,303 | 1,655 | 1,181 | 733 | 629 |
| E2&T.20% | Dominant | Dominant | Dominant | Dominant | Dominant | Dominant | Dominant | Dominant | Dominant | Dominant | Dominant | Dominant | Dominant | Dominant | Dominant | Dominant |

*Note: E1: Annual screening, E2: Biannual screening, E2 until 60+ E1: Biannual screening until 60 years old then annual screening until died, E2 until 55+ E1: Biannual screening until 55 years old then annual screening until died, E1&T.20%: Annual screening combined with increasing coverage of treatment by 20%, E2&T.20%: Biannual screening combined with increasing coverage of treatment by 20%, CVD: Cardiovascular disease.
