# Supplementary figures and images for: Cost-Effectiveness Analysis of Screening for and Managing Identified Hypertension for Cardiovascular Disease Prevention in Vietnam
Source: PLoS One. 2016 May 18;11(5):e0155699. doi: 10.1371/journal.pone.0155699 (PMC4871542; doi:10.1371/journal.pone.0155699)

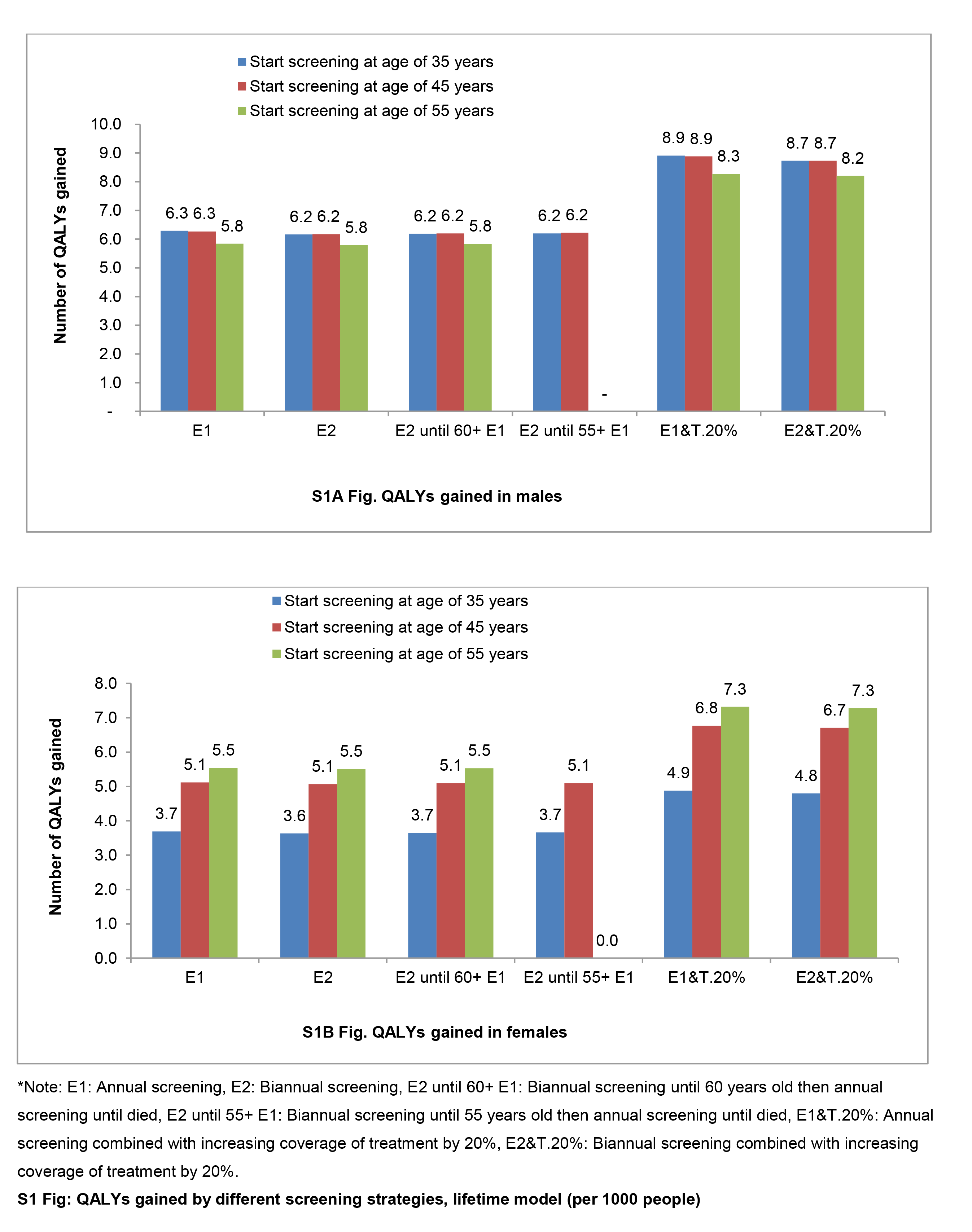

Supplement: S1 Fig — (TIF) [file pone.0155699.s005.tif]

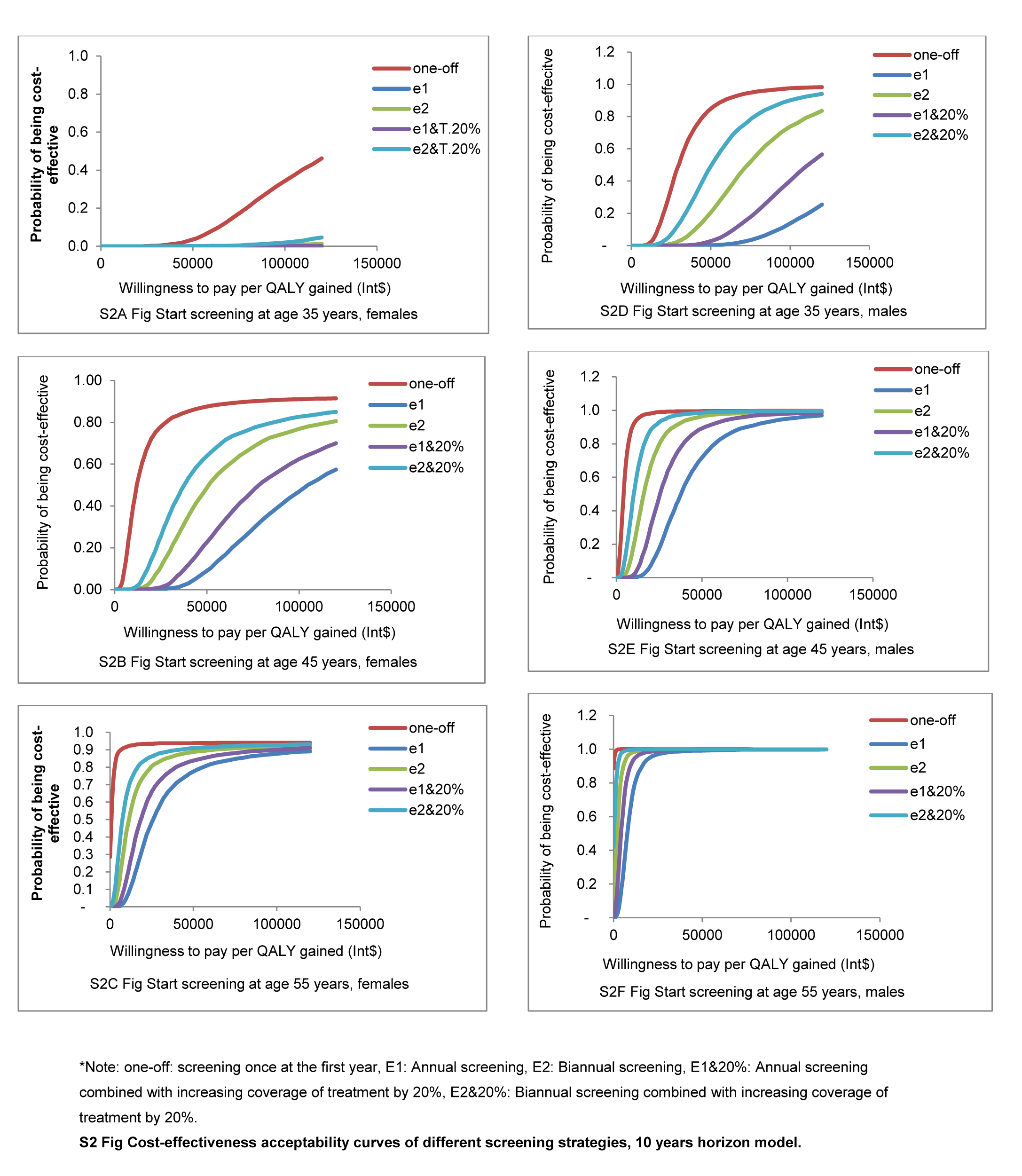

Supplement: S2 Fig — (TIF) [file pone.0155699.s006.tif]
